# An evaluation of R^2 ^as an inadequate measure for nonlinear models in pharmacological and biochemical research: a Monte Carlo approach

**DOI:** 10.1186/1471-2210-10-6

**Published:** 2010-06-07

**Authors:** Andrej-Nikolai Spiess, Natalie Neumeyer

**Affiliations:** 1 Department of Andrology, University Hospital Hamburg-Eppendorf, Hamburg, Germany; 2 Department of Mathematics, University of Hamburg, Hamburg, Germany

## Abstract

**Background:**

It is long known within the mathematical literature that the coefficient of determination R^2 ^is an inadequate measure for the goodness of fit in nonlinear models. Nevertheless, it is still frequently used within pharmacological and biochemical literature for the analysis and interpretation of nonlinear fitting to data.

**Results:**

The intensive simulation approach undermines previous observations and emphasizes the extremely low performance of R^2 ^as a basis for model validity and performance when applied to pharmacological/biochemical nonlinear data. In fact, with the 'true' model having up to 500 times more strength of evidence based on Akaike weights, this was only reflected in the third to fifth decimal place of R^2^. In addition, even the bias-corrected R^2^_adj _exhibited an extreme bias to higher parametrized models. The bias-corrected AICc and also BIC performed significantly better in this respect.

**Conclusion:**

Researchers and reviewers should be aware that R^2 ^is inappropriate when used for demonstrating the performance or validity of a certain nonlinear model. It should ideally be removed from scientific literature dealing with nonlinear model fitting or at least be supplemented with other methods such as AIC or BIC or used in context to other models in question.

## Background

Fitting nonlinear models to data is frequently applied within all fields of pharmaceutical and biochemical assay quantification. A plethora of nonlinear models exist, and chosing the right model for the data at hand is a mixture of experience, knowledge about the underlying process and statistical interpretation of the fitting outcome. While the former are of somewhat individual nature, there is a need in quantifying the validity of a fit by some measure which discriminates a 'good' from a 'bad' fit. The most common measure is the coefficient of determination R^2 ^used in linear regression when conducting calibration experiments for samples to be quantified [1]. In the linear context, this measure is very intuitive as values between 0 and 1 give a quick interpretation of how much of the variance in the data is explained by the fit. Although it is known now for some time that R^2 ^is an inadequate measure for nonlinear regression, many scientists and also reviewers insist on it being supplied in papers dealing with nonlinear data analysis. Several initial and older descriptions for R^2 ^being of no avail in nonlinear fitting had pointed out this issue but have probably fallen into oblivion [2-8]. This observation might be due to differences in the mathematical background of trained statisticians and biochemists/pharmacologists who often apply statistical methods but lack detailed statistical insight.

We made the observation that R^2 ^is still frequently being used in the context of performance or validity of a certain model when fit to nonlinear data. R^2 ^is not an optimal choice in a nonlinear regime as the the total sum-of-squares (TSS) is not equal to the regression sum-of-squares (REGSS) plus the residual sum-of-squares (RSS), as is the case in linear regression, and hence it lacks the above interpretation (see Additional File 1, paragraphs 1 & 2). To our observation, there is still a high occurrence in the present literature of all biomedical fields where the validity of nonlinear models is based solely on R^2 ^values, which might be a result of authors or reviewers not being aware of this fallacy. Additionally, almost all of the commercially available statistical software packages (i.e. Prism, Origin, Matlab, SPSS, SAS) calculate R^2 ^values for nonlinear fits, which is bound to unintentionally corroborate its frequent use. A further example is the TableCurve2 D software (Systat, USA) which can fit hundreds of nonlinear models to a given dataset automatically and then rank these by means of R^2^. Noted 25 years ago by Kvalseth [8], the user is usually not able to identify which of the eight different definitions of R^2 ^that are commonly being used in the literature is chosen for the analysis output in statistical software (see Additional File 1, Remark 4).

We thus aimed to point out the low performance of R^2 ^and its inappropriateness for nonlinear data analysis by basing our analysis on an extensive Monte Carlo simulation approach. This approach has fundamental advantages in the analysis of nonlinear data analysis [9] and can reveal tendencies within statistical methods by supplying the models and measures in question with thousands of generated datasets.

## Methods

### Creation of the 'true' model

In a first step, we fitted a three-parameter log-logistic model (L3, see Formula 3 below) by nonlinear least-squares to sigmoidal data that was taken from quantitative real-time polymerase chain reaction (qPCR). This yielded a sigmoidal model with the parameters b = -9.90, d = 11.07 and e = 24.75. We used the fitted values of this model and x-values from 10-35 as the 'true' model with sample size n = 26 for the following Monte Carlo simulation. This essentially gave a sigmoidal curve that can be encountered in many different areas of pharmacological/biochemical analysis. Specific to qPCR data, the x-values ("Cycles") are equidistant and not on a log-scale, as often encountered in dose-response analysis. For mathematical details, see Remark 7 in Additional File 1.

### Perturbation of data (Monte Carlo Simulation)

Using the fitted values as above, all datapoints were perturbed 2000 times by adding six different magnitudes (very low to high) of homoscedastic noise from a gaussian distribution with mean = 0 and standard deviations = 0.01, 0.02, 0.05, 0.1, 0.2 or 0.4. The noise of the data was therefore between 0.1% and 4% of the data range. This way, for each of six different settings (determined by different standard deviations), we obtained 2000 new data sets of sample size n = 26 with true model L3. For each of these data sets, nine different sigmoidal models differing in model type and number of parameters (Formulas 1-9) were fit. For mathematical details, see Remark 7 in Additional File 1.

**Formula 1: **Five parameter log-logistic model (L5):

**Formula 2: **Four-parameter log-logistic model (L4):

**Formula 3: **Three-parameter log-logistic model (L3):

**Formula 4: **Five parameter logistic model (B5):

**Formula 5: **Four-parameter logistic model (B4):

**Formula 6: **Three-parameter logistic model (B3):

**Formula 7: **Four-parameter Weibull model (W4):

**Formula 8: **Three-parameter Weibull model (W3):

**Formula 9: **Five-parameter baroreflex model (baro5):

### Calculation of measures for goodness-of-fit

For each simulation, we calculated the following measures for goodness-of-fit:

R^2^, using the most general definition [5,8]:

Formula 10:

with *RSS *= residual sum-of-squares, *TSS *= total sum-of-squares, *y *= response values,  = fitted values and  = the mean of response values. For a more detailed description see Remarks 1-6 in Additional File 1.

We chose to use the adjusted R^2 ^to compensate for possible bias due to different number of parameters:

Formula 11:

with *n *= sample size and *p *= number of parameters.

The Akaike Information Criterion (AIC, [10-12]), a measure that is widely accepted for measuring the validity within a cohort of nonlinear models and frequently used for model selection [13].

Formula 12:

with *p *= number of parameters and ln(*L) *= maximum log-likelihood of the estimated model. The latter, in the case of a nonlinear fit with normally distributed errors [13], is calculated by

Formula 13:

with *x_1_*, ..., *x_n _*= the residuals from the nonlinear least-squares fit and *N *= their number.

To provide a fair playing ground, we employed an AIC variant that corrects for small sample sizes, the bias-corrected AIC (AICc):

Formula 14:

with *n *= sample size and *p *= number of parameters.

In order to obtain values for the validity of a fit, we used Akaike weights which calculate the *weight of evidence *for each model within a cohort of models in question [12-14]:

Formula 15:

with *i*, *k *= model numbers, Δ_i_(*AIC*) = the difference in AIC of each model in comparison to the model with the lowest AIC, subsequently normalized to their sum (denominator).

Also here, we used the bias-corrected AICc for calculating the Akaike weights.

We also chose to employ the Bayesian Information Criterion (BIC), which gives a higher penalty on the number of parameters [15]:

Formula 16:

with *p *= number of parameters, *n *= sample size and *L *= maximum likelihood of the estimated model.

Furthermore, the residual variance as the part of the variance that cannot be accounted for by the model:

Formula 17:

with *RSS *= residual sum-of-squares, *n *= sample size and *p *= number of parameters.

The variance of a least-squares fit is also characterized by the chi-square statistic defined as **Formula 18:**

where *y_i _*= response values, *f(x_i_) *= the fitted values and  = the uncertainty in the individual measurements *y_i_*. We further define the reduced chi-square as a useful measure [16] by

Formula 19:

with *ν *= *n - p *(degrees of freedom). If the fitting function is a good approximation to the parent function, then the variances of both should agree well, and the reduced chi-square should be approximately unity. If the reduced chi-square is much larger than 1 (i.e. 10 or 100), it means that one is either overly optimistic about the measurement errors or that one selected an inappropriate fitting function. If reduced chi-square is too small (i.e. 0.1 or 0.01) it may mean that one has been too pessimistic about measurement errors. For this work, models were selected based on reduced chi-square by being closest to 1.

### Analysis of the simulation data

Two different approaches were pursued within the Monte Carlo simulated data. To reveal general tendencies, we averaged the values of R^2^_adj_, AICc, BIC, residual variance and reduced chi-square from all 2000 simulated data sets (for each noise magnitude and each of the 9 models). To permit a more detailed insight for each simulation and to compare the measures on the single model level, we selected the best model in each iteration based on the highest R^2^_adj_, lowest AICc, lowest BIC, lowest residual variance and smallest difference to unity for reduced chi-square. For the latter, we used as the measurement uncertainty  the *a priori *known squared standard deviation from the homoscedastic noise that was applied to produce the random data of the Monte Carlo simulations.

Finally, we calculated the percentage of selecting the 'true' model L3 in all iterations.

### Code for the simulations

All simulations were conducted using *R*, a well reputed and open-source statistical programming language [17]. Nonlinear curve fitting was done by using functionality from the R package *qpcR *[18]. The commented code for the simulations can be obtained from Additional File 2.

## Results and Discussion

**Figure 1:**
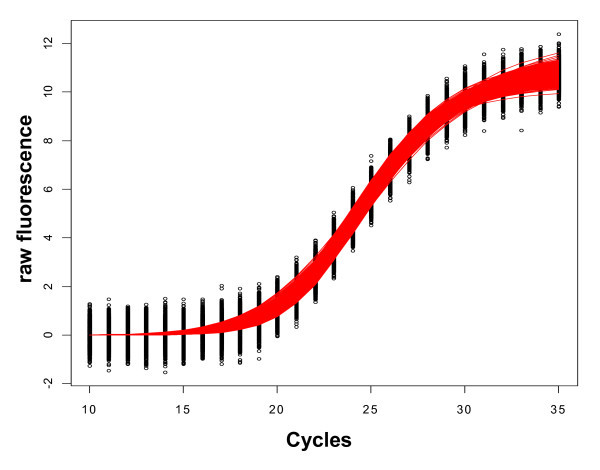
**Graph illustrating the noise model used for the simulations**. 2000 simulations of random gaussian noise with mean = 0 and s.d. = 0.4 were added to the fitted values of a three-parameter log-logistic model (L3) fit to real-time quantitative PCR data. This resulted in the point cloud (black dots) of response values and the band of red lines reflecting the fitted curves of all simulations with the same model L3 applied.

Figure 1 illustrates the simulated data that was used as the basis of our analysis. Starting from the fitted values of a three-parameter log-logistic model (L3), different amounts of homoscedastic gaussian noise were added to the fitted values resulting in the point clouds as shown. We analyzed six different magnitudes of gaussian noise in total, from low noise (s.d. = 0.01, 0.02), medium noise (s.d. = 0.05, 0.1) to high noise (s.d. = 0.2, 0.4; see Figure 1) with a total of 2000 simulations per noise setup.

**Figure 2:**
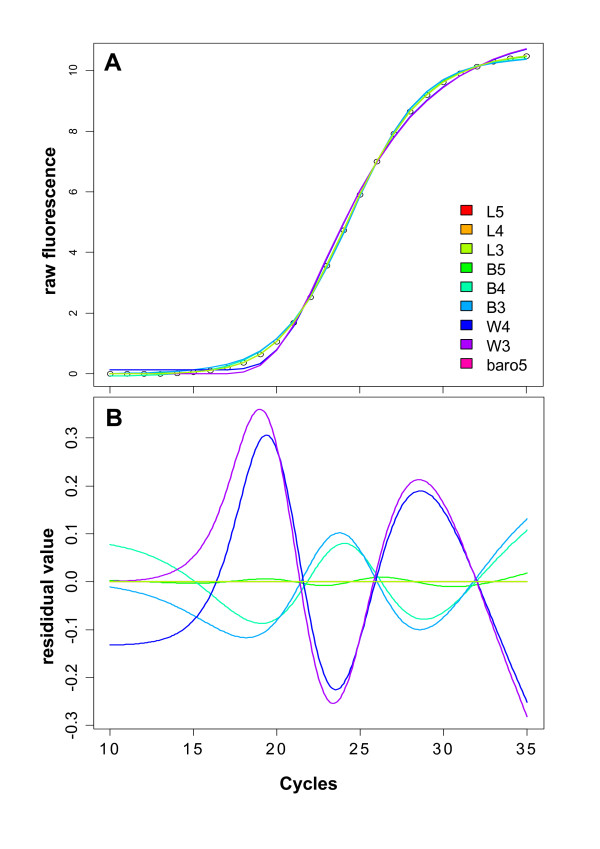
**Performance of the nine different sigmoidal models on the fitted values from a three-parameter log-logistic model**. (A) The nine different sigmoidal models were fit by nonlinear least-squares to the fitted data from the L3 model. (B) Residual plot depicting the performance of each of the nine models in respect to fitting to the data from the L3 model. As expected, model L3 (light green, reference curve) has zero residual value as having been fit to the data obtained from the same model. Several other models also fit the data exceptionally well (L5, L4, baro5) and are not visible due to being overlayed by the L3 curve. Descriptions for the abbreviated models can be found under Formulas 1-9.

Fitting all nine different sigmoidal models to the fitted values of the 'true' model (L3) is depicted in Figure 2A, demonstrating the differences against this model when noise is completely lacking. All logistic models fit well in this context, which tallies with the observation of five-parameter models exhibiting increased performance due to accomodating asymmetrical structures [19]. Visualized also by a residuals plot that delivers higher resolution of the residuals (Figure 2B), the log-logistic models (L3, L4, L5) provide very small residual values, the logistic models (B3, B4, B5) have higher residual values and the Weibull models (W3, W4) are significantly inferior. This is evident especially in the upper and lower region from the point of inflection.

**Figure 3:**
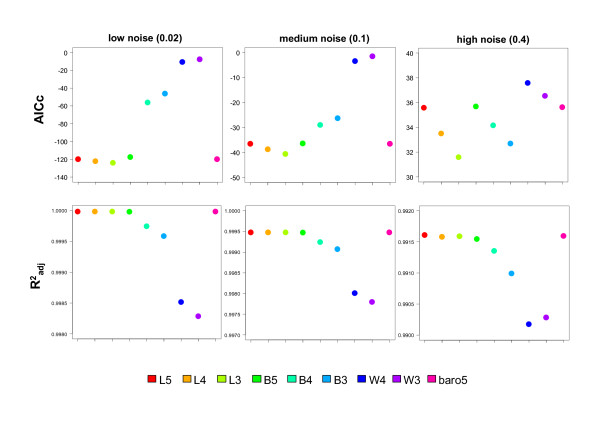
**Analysis of adjusted R^2 ^and corrected AIC of nine different sigmoidal models on fitted data from a three-parameter log-logistic model (L3)**. Three different magnitudes of homoscedastic gaussian noise (low: 0.02; medium: 0.1; high: 0.4) were added to the fitted data (2000 simulations), each of the nine sigmoidal model fit by nonlinear least-squares and the two measures collected for each simulation. Finally, the measures were averaged and displayed as point graphs. Upper panel: AICc, lower panel: R^2^_adj_. Descriptions for the abbreviated models can be found under Formulas 1-9. Coefficients of variation for all simulations were below 5% and hence omitted. More detailed data for the measures can be found in Table 1.

AICc and adjusted R^2^_adj _were compared by averaging the output of all 2000 simulations and for three different magnitudes (low, middle, high) of homoscedastic gaussian noise (Figure 3). Not surprisingly, the R^2^_adj _values decrease and the AICc values increase with higher noise (left to right). But the major problem in the use of R^2 ^is clearly evident within the simulation setup: the AIC differences (delta-AIC) between some models can be 78 (compare L3 and B3 models at s.d. = 0.02), which when transferred to Akaike weights result in a *weight of evidence *16 (!) orders of magnitude in favor of L3. One might be inclined to say that this is major evidence for the first model being in favor of the second one, but in respect to the corresponding R^2^_adj _values, only the fourth decimal place is affected. This tendency is also found for higher noise, despite AICc values increasing and R^2^_adj _values decreasing. Even in the presence of relatively high noise (s.d. = 0.4), at least for the simulation setup in this work, R^2^_adj _would hardly drop below 0.99. Comparing the Akaike weight of the models with the R^2^_adj _values in Table 1 shows the strong discrepancy in scale changes of these two measures when comparing a cohort of different models.

**Table 1:** Summary of the Monte-Carlo simulation study.

s.d = 0.01	Model	R^2^_adj_	AICc	Akaike weights	BIC	resVar	red. Chi^2^
	**L5**	0.99999471	-156.22	0.0818	-156.35	0.00009933	0.9924
	**L4**	0.99999471	-158.36	0.2389	-158.33	0.00009935	0.9930
	**L3**	0.99999470	-160.20	0.5969	-160.33	0.00009959	0.9927
	**B5**	0.99999258	-147.31	0.0010	-147.44	0.00013945	1.4739
	**B4**	0.99975702	-57.72	0.0000	-57.69	0.00456696	42.5939
	**B3**	0.99959911	-47.29	0.0000	-47.42	0.00734455	63.5805
	**W4**	0.99853046	-10.89	0.0000	-10.85	0.02762048	282.3939
	**W3**	0.99829856	-7.91	0.0000	-8.04	0.03336157	334.1353
	**baro5**	0.99999471	-156.21	0.0814	-156.34	0.00009936	0.9927
							
**s.d = 0.02**	**L5**	0.99997869	-119.98	0.0774	-120.11	0.00040059	0.9946
	**L4**	0.99997868	-122.13	0.2273	-122.10	0.00040068	0.9959
	**L3**	0.99997871	-124.06	0.5954	-124.19	0.00040007	0.9957
	**B5**	0.99997653	-117.49	0.0224	-117.62	0.00044108	1.1135
	**B4**	0.99974038	-56.14	0.0000	-56.11	0.00487955	11.4098
	**B3**	0.99958227	-46.30	0.0000	-46.43	0.0076527	16.6958
	**W4**	0.99851513	-10.64	0.0000	-10.61	0.02790803	71.2971
	**W3**	0.99828395	-7.71	0.0000	-7.84	0.03364791	84.1649
	**baro5**	0.99997869	-119.98	0.0775	-120.11	0.00040044	0.9956
							
**s.d. = 0.05**	**L5**	0.99986676	-72.33	0.0765	-72.46	0.00250459	0.9897
	**L4**	0.99986656	-74.44	0.2194	-74.41	0.00250829	0.9904
	**L3**	0.99986662	-76.34	0.5674	-76.47	0.00250720	0.9882
	**B5**	0.99986439	-71.87	0.0608	-72.00	0.00254924	1.0096
	**B4**	0.99962966	-47.44	0.0000	-47.40	0.00696139	2.6343
	**B3**	0.99946832	-40.44	0.0000	-40.57	0.00973888	3.4859
	**W4**	0.99839915	-8.85	0.0000	-8.81	0.03009194	12.3020
	**W3**	0.99817327	-6.21	0.0000	-6.34	0.03582530	14.3447
	**baro5**	0.99986669	-72.32	0.0759	-72.45	0.00250592	0.9902
							
**s.d. = 0.1**	**L5**	0.99947371	-36.57	0.0742	-36.70	0.00989448	0.9984
	**L4**	0.99947362	-38.73	0.2180	-38.70	0.00989607	0.9972
	**L3**	0.99947374	-40.62	0.5618	-40.75	0.00989674	0.9972
	**B5**	0.99947135	-36.46	0.0701	-36.59	0.00993888	1.0037
	**B4**	0.99923746	-29.03	0.0017	-28.99	0.01433573	1.4052
	**B3**	0.99907025	-26.35	0.0004	-26.48	0.01703305	1.6200
	**W4**	0.99800791	-3.50	0.0000	-3.47	0.03745226	3.8282
	**W3**	0.99779177	-1.55	0.0000	-1.68	0.04333951	4.3370
	**baro5**	0.99947355	-36.56	0.0737	-36.69	0.00989740	0.9982
							
**s.d. = 0.2**	**L5**	0.99786138	-0.07	0.0675	-0.20	0.04025347	0.9948
	**L4**	0.99785879	-2.18	0.1942	-2.15	0.04030142	0.9940
	**L3**	0.99785563	-4.04	0.4930	-4.17	0.04035658	0.9928
	**B5**	0.99785959	-0.05	0.0668	-0.18	0.04028754	0.9960
	**B4**	0.99762513	0.49	0.0512	0.52	0.04470201	1.1027
	**B3**	0.99740761	0.20	0.0592	0.06	0.04751535	1.1543
	**W4**	0.99640798	11.41	0.0002	11.44	0.06760434	1.6764
	**W3**	0.99625943	11.71	0.0002	11.58	0.07350697	1.8059
	**baro5**	0.99786149	-0.07	0.0676	-0.20	0.04025146	0.9941
							
**s.d. = 0.4**	**L5**	0.99160836	35.58	0.0490	35.45	0.15887711	0.9987
	**L4**	0.99157911	33.50	0.1387	33.53	0.15941969	0.9981
	**L3**	0.99158878	31.58	0.3613	31.45	0.15928956	0.9980
	**B5**	0.99154493	35.68	0.0466	35.55	0.15991031	1.0001
	**B4**	0.99135309	34.16	0.0996	34.19	0.16370200	1.0242
	**B3**	0.99098926	32.68	0.2084	32.55	0.16621846	1.0372
	**W4**	0.99017401	37.58	0.0180	37.61	0.18602148	1.1663
	**W3**	0.99028174	36.53	0.0305	36.40	0.19208006	1.1995
	**baro5**	0.99159379	35.62	0.0480	35.49	0.15915529	0.9989

Six different magnitudes of gaussian noise (low: s.d. = 0.01, 0.02; medium: s.d. = 0.05, 0.1; high: s.d. = 0.2, 0.4) were added to the fitted data of a three-parameter log-logistic model (L3). Nine different sigmoidal models were fit by nonlinear least-squares to the perturbed data and different measures for the goodness of fit (see Materials & Methods) averaged after all 2000 iterations. From the AICc values, Akaike weights were calculated in order to obtain the *weight of evidence *of the models.

However, differences in scale changes would be unimportant if the direction of change is always reciprocally (i.e. a lower AICc always corresponding with a higher R^2^_adj_). As can be deduced from Table 1, this is not always the case. Using the averaged measures from the simulations, R^2^_adj _and AICc did not always behave reciprocally. For instance, at s.d. = 0.05, 0.2 and 0.4 the 'true' model L3 has a lower AICc than L5 (and ~ 8 times more *weight of evidence *by Akaike weights), but R^2^_adj _values are also lower in the fifth to eighth decimal place. Using R^2^_adj _for model selection would therefore have resulted in a model which is clearly not in favor based on Akaike weights.

On the averaged values, BIC essentially shows the same characteristics as AICc. Interestingly, the *residual variance *is higher in the 'true' model L3 than L5 for most noise regimes (although also only in the third to eighth decimal place), indicating this to be a relatively unfavorable measure. Likewise, the reduced chi-square exhibited a tendency to be closest to unity for higher parameter models (L4, L5, B5) with increasing noise, here also only affected in the third decimal place.

**Figure 4:**
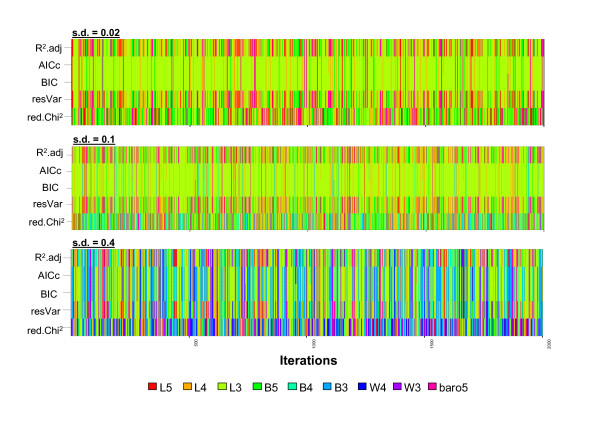
**Analysis of model selection bias between for different measures of goodness-of-fit**. Three different magnitudes of homoscedastic gaussian noise (0.02%; 0.1%; 0.4%) were added to the fitted data of model L3. The nine different sigmoidal models were fit and the different measures for goodness-of-fit collected at each iteration. The best model was selected for each measure and displayed for all iterations as a coloured selection heatmap. Light green reflects the 'true' model L3.

To acquire more detailed insight into the performance of the different measures in respect to the selection of the best model and in dependence of different noise magnitudes, we selected the best model of each iteration by each of the measures. This approach can reveal features that are not evident when calculating the averaged measures of all simulations. We summarized the outcome of this analysis as a heatmap display in Figure 4 and as 'model selection frequency' in Table 2. Within each image plot, the selected models are shown with the same colour coding as in Figures 1, 2 and 3. For the low (s.d. = 0.02) and medium (s.d. = 0.1) setup, R^2^_adj_, residual variance (resVar) and reduced chi-square performed not optimally, selecting the true model L3 only in 28-43% of the iterations. Both measures exhibited a severe bias in the selection of models with a higher number of parameters (L4, L5, B5, baro5). It is interesting to note that although R^2^_adj _and the residual variance 'correct' against the number of parameters, there is no positive effect on the ability to select the 'true' model. This may be due largely to the setup in this work which features a relatively high sample size (*n *= 26) compared to the number of parameters (p = 3-5) and leaves the denominator *n - p *in both measures relatively unaffected. In contrast, both AICc and BIC performed superior in the selection of the 'true' model L3 at these magnitudes of noise with over 80% of all iterations, but with a slight bias to models with a lower number of parameters at medium noise. At high noise (s.d. = 0.4, corresponding to 4% noise of the data range) the performance of all measures decreased markedly, most probably from the effect of the simulated data losing the structural features typical of the L3 model when high noise is added. Despite this negative trend, AICc and BIC displayed increased performance even at high noise. The two measures selected also a significant number of iterations (about 40%) of other models with the same number of parameters (B3, W3), which might be an indication for a small bias to lower parametrized models. Consequently, and based on the analysis of a sigmoidal nonlinear setup as described here, we feel compelled to give the following summary:

**Table 2:** Model selection frequency for the different measures of goodness-of-fit.

	Model	R^2^adj	AICc	BIC	resVar	red. Chi^2^
**s.d. = 0.02**	**L5**	228	40	41	231	227
	**L4**	341	170	154	347	261
	**L3**	867	1648	1660	857	567
	**B5**	305	90	91	304	689
	**B4**					
	**B3**					
	**W4**					
	**W3**					
	**baro5**	259	52	54	261	256
**L3 [%]**		43.4	82.4	83.0	42.9	28.4
						
**s.d. = 0.1**	**L5**	124	10	10	122	118
	**L4**	349	161	145	365	288
	**L3**	958	1668	1684	939	576
	**B5**	279	30	30	283	308
	**B4**	35	53	51	34	361
	**B3**	5	43	44	6	144
	**W4**					
	**W3**					
	**baro5**	250	35	36	251	205
**L3 [%]**		47.9	83.4	84.2	47.0	28.8
						
**s.d. = 0.4**	**L5**	42	5	6	43	69
	**L4**	234	72	65	243	187
	**L3**	698	982	989	667	374
	**B5**	55	3	3	61	67
	**B4**	257	81	76	194	208
	**B3**	303	575	582	406	335
	**W4**	51	46	43	89	305
	**W3**	221	226	226	145	357
	**baro5**	139	10	10	152	98
**L3 [%]**		34.9	49.1	49.5	33.4	18.7

Same setup as in Table 1 but summarizing the model selection for each iteration and each measure by selection frequency. A percentage of selecting the 'true' model L3 is given as a summary.

1) The use of highly inferior nonlinear models is reflected only in the third or fourth decimal place of R^2 ^and thus the description of single models when using R^2 ^is not meaningful, as this measure tends to be uniformly high when a set of models is inspected. This has also been noted by others [20]. Additionally, R^2 ^and even its 'bias-corrected' counterpart R^2^_adj _are severely biased in favor of models with more parameters when it comes to model selection. The same accounts for the residual variance, which is also commonly used. AICc and BIC do not exhibit this bias and provide a much clearer picture and improved performance when it comes to selecting the 'true' model. The Akaike weights are especially useful in obtaining an overview of the *weight of evidence *of one model over the other, which is impossible with the *per se *high R^2 ^values. This approach requires anyhow that it is mandatory to supply several models in question.

2) In a background of low and medium experimental noise, R^2^_adj _and AICc selected different models with AICc selecting the 'true model' twice as often (82.2% versus 43%). This finding emphasizes the importance of this measure in nonlinear model selection. At a high noise level rarely encountered in the modelling of pharmacological/biochemical data (4% of data range), AICc still performed superior to R^2^_adj_. These results tempt us to conclude that the *degree of freedom *term *n - p *in the denominator of the residual variance and R^2^_adj _is not sufficient alone to compensate the effect of the number of increasing parameters. The same seems to be the case for the reduced chi-square, which is also frequently used for model selection purposes.

In this work we show that R^2 ^is an inappropriate measure when used in the field of nonlinear fitting. Efforts have been made to develop R^2^-like measures for the most common nonlinear regression models [21], but here we focused on the inadequateness of its use by using a data perturbation approach and comparing its performance in comparison to AICc, BIC, residual variance and reduced chi-square. Model selection in nonlinear statistical literature is usually divided into the frequentist methods, for example F-tests on the residual variance that are restricted to nested models [13], or measures from information theory such as AIC which are often used to compare non-nested models. Indeed, it has been shown that the latter approach can often perform better than F-tests [22]. In the field of biochemical and pharmacological literature there is a reasonably high occurrence in the use of R^2 ^as the basis of arguing against or in favor of a certain model. As a result from this work, we would like to advocate that R^2 ^should not be reported or demanded in phamacological/biochemical literature when discussing nonlinear data analysis. Authors as well as reviewers should be aware that demonstrating the validity of single nonlinear models by using solely R^2 ^is not state-of-the-art and should be replaced or supplemented by AIC/AICc/BIC values (or their corresponding *weights of evidence*) of several possible models that are in question. The latter will give the dedicated reader the possibility to obtain information about how much a certain model is in favor over others, a feature that will not be evident by minor changes in R^2 ^which tends to be uniformly high and is rarely affected more than in the third or fourth decimal place.

## Conclusions

Although frequently being used in the present pharmocological/biochemical literature for describing the validity of a nonlinear fit, R^2 ^is an unfavorable measure that is rarely affected more than in the third or fourth decimal place, even in scenarios with highly inferior models. Our Monte Carlo simulations have shown that AIC, AICc or BIC perform significantly better in this respect so that authors as well as reviewers should be aware of this issue and refrain from using or asking for R^2 ^values when nonlinear models are under investigation.

## Competing interests

The authors declare that they have no competing interests.

## Authors' contributions

ANS conducted the simulations and wrote the manuscript. NN helped in drafting the manuscript and developed the statistical workflow. All authors read and approved the final manuscript.

## Supplementary Material

Additional File 1 **Mathematical derivation and concise discussion of features and pitfalls in the use of R^2 ^in nonlinear regression and description of the simulation setup**. Click here for file

Additional File 2 ***R *code used for conducting the simulations. 'pcrsim' of package 'qpcR' is the workhorse function that creates simulated data starting from the fitted value, adding a desired noise structure and testing different sigmoidal models on the perturbed data. 'code' collects the results and summarizes the data as shown in this manuscript**. R script file for the *R *statistical environment. Click here for file
