# Supplementary material for: An evaluation of R2 as an inadequate measure for nonlinear models in pharmacological and biochemical research: a Monte Carlo approach
Source: BMC Pharmacol. 2010 Jun 7;10:6. doi: 10.1186/1471-2210-10-6 (PMC2892436; doi:10.1186/1471-2210-10-6)
Supplement: Additional File 1 — Mathematical derivation and concise discussion of features and pitfalls in the use of R2 in nonlinear regression and description of the simulation setup. [file 1471-2210-10-6-S1.PDF]

Supplemental Data 1

"A re-evaluation of R^2 as an inadequate measure for nonlinear models in pharmacological and biochemical research: A Monte Carlo approach"

by Andrej-Nikolai Spiess & Natalie Neumeyer

Let independent data (X_i, Y_i), $i = 1, \ldots, n$, be observed from the true model

$$Y \;=\; f(X) + \varepsilon \tag{$*$}$$

with unknown regression function f and noise variance $\sigma^2 = \mathrm{Var}(\varepsilon)$ under the usual assumptions. Assume we fit a regression model

$$Y \;=\; f_\vartheta(X) + \varepsilon, \quad \vartheta \in \Theta$$

by least-squares, which gives an estimator $\hat\vartheta$ for the unknown parameter. This least squares estimator $\hat\vartheta$ is obtained by minimizing the residual sum of squares $RSS = \sum_{i=1}^n (Y_i - f_\vartheta(X_i))^2$ and estimates the value ϑ that minimizes

$$E[(f(X) - f_\vartheta(X))^2]$$

over $\vartheta \in \Theta$. We only fit the correct model if there exists some $\vartheta \in \Theta$ such that $f = f_\vartheta$. From the fit we obtain the fitted values $\hat Y_i = f_{\hat\vartheta}(X_i)$, $i = 1, \ldots, n$, and define the total sum of squares (TSS), residual sum of squares (RSS) and regression sum of squares ($REGSS$) as

$$TSS = \sum_{i=1}^n \left(Y_i - \overline{Y}\right)^2, \quad RSS = \sum_{i=1}^n \left(Y_i - \hat Y_i\right)^2, \quad REGSS = \sum_{i=1}^n \left(\hat Y_i - \overline{\hat Y}\right)^2. \tag{1}$$

Note that in linear models one has $\overline{\hat Y} = \overline{Y}$ and then $REGSS$ is often defined as $\sum_{i=1}^n (\hat Y_i - \overline{Y})^2$.

The most common definition of the nonlinear coefficient of determination is

$$R^2 = 1 - \frac{RSS}{TSS}. \tag{2}$$

For an increasing sample size $n \to \infty$ the coefficient R^2 estimates the following term (see Remark 6 below for the derivation),

$$r^2 \;=\; 1 - \frac{E[(f(X) - f_\vartheta(X))^2] + \sigma^2}{\mathrm{Var}(f(X)) + \sigma^2} = \frac{\mathrm{Var}(f(X)) - E[(f(X) - f_\vartheta(X))^2]}{\mathrm{Var}(f(X)) + \sigma^2}. \tag{3}$$

Remark 1. If the true model is fitted, i. e. if we have $f_\vartheta = f$, then asymptotically ($n \to \infty$) we have

$$r^2 = \frac{\mathrm{Var}(f(X))}{\mathrm{Var}(f(X)) + \sigma^2}. \tag{4}$$

Remark 2. From formula (4) we see that R^2 is sensitive to changes in the variance σ^2 of the noise, even if we fit the true model. This can result in misinterpretations. However, as can be seen from (3),

r^2 gets larger with a better fit (i. e. smaller values of $E[(f(X) - f_\vartheta(X))^2]$) and therefore indeed could be used for comparison of models. But its value is difficult to interpret and can even be negative.

Remark 3. The measure R^2 compares the performed fit with the fit available by a constant regression function (the "neutral standard" estimated by the mean $\overline{Y}$). For observations obtained from sigmoidal models as considered in the paper at hand that are far from a constant model, R^2 resulted in values close to 1, even for incorrect models. A large value of R^2 should therefore not be misinterpreted as indication of a correct model fit.

Remark 4. One should be even more careful when using the coefficient of determination from statistical software, when it is not clear how R^2 is calculated. Sometimes the following alternative definitions are used,

$$R_1^2 = \frac{REGSS}{TSS}, \quad R_2^2 = \frac{REGSS}{REGSS + RSS} \tag{5}$$

which for an increasing sample size $n \to \infty$ estimate the following terms, respectively, (see Remark 6 below for the derivation)

$$r_1^2 \;=\; \frac{\mathrm{Var}(f_\vartheta(X))}{\mathrm{Var}(f(X)) + \sigma^2}, \qquad r_2^2 \;=\; \frac{\mathrm{Var}(f_\vartheta(X))}{\mathrm{Var}(f_\vartheta(X)) + E[(f(X) - f_\vartheta(X))^2] + \sigma^2}. \tag{6}$$

We see from formula (6) that both R_1^2 and R_2^2 should not be used to compare models. A measure suitable to compare models (with respect to the least squares criterion) should give larger values when $E[(f(X) - f_\vartheta(X))^2]$ gets smaller by choice of a better model $\{f_\vartheta | \vartheta \in \Theta\}$. Neither r_1^2 nor r_2^2 fulfill that requirement (for r_2^2 note that with the change of the model also $\mathrm{Var}(f_\vartheta(X))$ may change). It can already be seen for finite sample sizes n from the definitions (2) and (5) that in general only R^2 gets smaller for a smaller RSS due to a change of the model. Moreover, R_1^2 can assume values larger than 1.

Remark 5. In linear models with regression function $f_\vartheta(X) = g(X)^T\vartheta$, where g is a known function, one has $TSS = REGSS + RSS$ and hence, $R^2 = R_1^2 = R_2^2$. But even then, R^2 is sensitive to changes in σ^2.

Remark 6. Derivation of formulas (3) and (6): Consider the definitions in (1), (2) and (5).
The term $\frac{1}{n}\sum_{i=1}^n (Y_i - \overline{Y})^2$ estimates $\mathrm{Var}(Y) = \mathrm{Var}(f(X)) + \sigma^2$.
Similarly, $\hat{Y}_i$ estimates $f_\vartheta(X_i)$, the term $\frac{1}{n}\sum_{i=1}^n (\hat{Y}_i - \overline{\hat{Y}})^2$ estimates $\mathrm{Var}(f_\vartheta(X))$.
Finally, $Y_i - \hat{Y}_i = f(X_i) - \hat{Y}_i + \varepsilon_i$ estimates $f(X_i) - f_\vartheta(X_i) + \varepsilon_i$, and hence, $\frac{1}{n}\sum_{i=1}^n (Y_i - \hat{Y}_i)^2$ estimates the expectation $E[(f(X) - f_\vartheta(X) + \varepsilon)^2] = E[(f(X) - f_\vartheta(X))^2] + \sigma^2$.

Remark 7. In the main article data were generated as follows. Original data were fitted with model L3 from Formula 3 in the main article. Those fitted values $\hat{y}$ (at covariate X) were then used as values $f(X)$ in model (*) and were perturbed by homoscedastic Gaussian noise ε with variance σ^2, which gave data y following model (*) with known true model L3. Different models were then fitted to those data.
